# P361: Biomedical waste management in hospitals: the case of large burned: the burn centre center of Abidjan

**DOI:** 10.1186/2047-2994-2-S1-P361

**Published:** 2013-06-20

**Authors:** R Oyourou, J Sissoko

**Affiliations:** 1 SAMU-CI, Great Burn Center, Abidjan, Côte d'Ivoire

## Introduction

Biomedical waste presents potential hazards for people who generate it in healthcare settings or individuals who handle or are exposed to it after its mismanagement.

## Objectives

To investigate the knowledge, attitudes and practices of medical waste management in Abidjan’s Burn Center.

## Methods

Prospective preliminary study type cross-sectional, made over a period of two weeks on the types of medical waste and the analysis of knowledge and practices of personnel. The study involved 13 nurses, 05 doctors 10 aides, and 2 maintenance agents.

## Results

The majority of the waste is needles and syringes, gauze, gloves, pouches blood, infusion sets, urine bags, tubes, vials of injectable ampoules cotton and pharmaceutical waste.

Regarding knowledge of risk medical waste: 96% know the risks of mismanagement of medical waste; there is no waste sorting prior to garbage collection. After dressing, the waste shall revert to the central waste bin are all set in the same trash bag and dumped without every day in the central bin. The needles are pre-stored in bottles. Proper equipment for better waste management is inadequate as 96% of respondents. Medical waste is collected, does not undergo any treatment and is transported to the landfill Akouedo a service provider. In addition, no health staff received training on the management of biomedical waste and no campaign has been carried out in the center.

## Conclusion

This study shows the need to strengthen the process of medical waste management in a burn center through education, training and the provision of adequate equipment.

## Disclosure of interest

None declared

